# Publisher Correction: A single-dose mRNA vaccine provides a long-term protection for hACE2 transgenic mice from SARS-CoV-2

**DOI:** 10.1038/s41467-021-22820-x

**Published:** 2021-04-15

**Authors:** Qingrui Huang, Kai Ji, Siyu Tian, Fengze Wang, Baoying Huang, Zhou Tong, Shuguang Tan, Junfeng Hao, Qihui Wang, Wenjie Tan, George F. Gao, Jinghua Yan

**Affiliations:** 1 CAS Key Laboratory of Microbial Physiological and Metabolic Engineering, Institute of Microbiology, Chinese Academy of Sciences, Beijing, China; 2 University of Chinese Academy of Sciences, Beijing, China; 3 MHC Key Laboratory of Biosafety, National Institute for Viral Disease Control and Prevention, China CDC, Beijing, China; 4 CAS Key Laboratory of Pathogenic Microbiology and Immunology, Institute of Microbiology, Chinese Academy of Sciences, Beijing, China; 5 Shanxi Academy of Advanced Research and Innovation, Taiyuan, China; 6 Core Facility for Protein Research, Institute of Biophysics, Chinese Academy of Sciences, Beijing, China; 7 Chinese Center for Disease Control and Prevention (China CDC), Beijing, China

**Keywords:** RNA vaccines, SARS-CoV-2

Correction to: *Nature Communications* 10.1038/s41467-021-21037-2, published online 03 February 2021.

The original version of the Supplementary information associated with this Article had Supplementary Figure 2 duplicated as Supplementary Figure 4. The HTML has been updated to include a corrected version of the Supplementary information.

## Supplementary information

Supplementary information

