# Supplementary material for: A single-dose mRNA vaccine provides a long-term protection for hACE2 transgenic mice from SARS-CoV-2
Source: Nat Commun. 2021 Feb 3;12:776. doi: 10.1038/s41467-021-21037-2 (PMC7858593; doi:10.1038/s41467-021-21037-2)
Supplement: Supplementary file 1 — Supplementary Information [file 41467_2021_21037_MOESM1_ESM.pdf]

Supplementary Information for:

A single-dose mRNA vaccine provides a long-term protection for hACE2 transgenic mice from SARS-CoV-2

Qingrui Huang[1,†], Kai Ji[1,2,†], Siyu Tian[1,2,†], Fengze Wang[1,2,†], Baoying Huang[3], Zhou Tong[4,5], Shuguang Tan[4], Junfeng Hao[6], Qihui Wang[1,4], Wenjie Tan[3]✉, George F. Gao[2,4,7]✉, Jinghua Yan[1,2,4]✉

[1] CAS Key Laboratory of Microbial Physiological and Metabolic Engineering, Institute of Microbiology, Chinese Academy of Sciences, Beijing 100101, China.

[2]University of Chinese Academy of Sciences, Beijing 101408, China.

[3]MHC Key Laboratory of Biosafety, National Institute for Viral Disease Control and Prevention, China CDC, Beijing 102206, China.

[4] CAS Key Laboratory of Pathogenic Microbiology and Immunology, Institute of Microbiology, Chinese Academy of Sciences, Beijing 100101, China.

[5] Shanxi Academy of Advanced Research and Innovation, Taiyuan 030032, China.

[6]Core Facility for Protein Research, Institute of Biophysics, Chinese Academy of Sciences, Beijing 100101, China.

[7]Chinese Center for Disease Control and Prevention (China CDC), Beijing 102206, China

[†]These authors contributed equally to this work.

[✉]Corresponding authors. Email: tanwj@ivdc.chinacdc.cn (Wenjie Tan); gaof@im.ac.cn (George F. Gao); yanjh@im.ac.cn (Jinghua Yan).

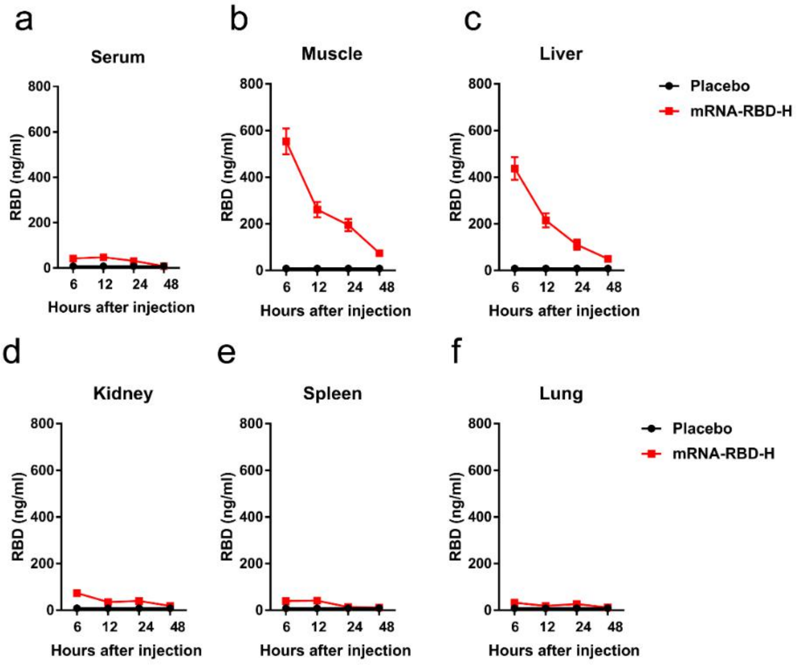

Supplementary Fig. 1 | Duration and distribution of RBD production from mRNA-RBD LNPs *in vivo*. Groups of BALB/c mice (n=16) were vaccinated with one injection of 15 μg mRNA-RBD or with a placebo. Four mice per group were euthanized at 6, 12, 24 and 48 h post injection. Serum (a), muscle (b), liver (c), kidney (d), spleen (e) and lung (f) samples of each mouse were harvested for quantitation of RBD expression level by ELISA. mRNA-RBD-H indicates the high-dose vaccine (15 μg). Data are means ± SEM (standard error of the mean). Placebo animals = black circles; mRNA-RBD-H-vaccinated animals = red squares. The data are representative of two independent experiments. Source data are provided as a Source Data file.

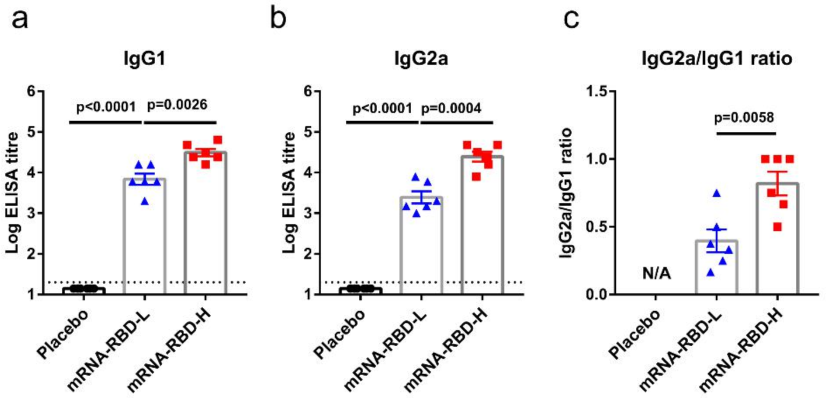

Supplementary Fig. 2 | A balanced Th1/Th2 immune response induced by mRNA-RBD. **Related to Fig. 2.** BALB/c mice (n=6) were immunized with one injection of mRNA-RBD at different doses or with a placebo. Sera were collected four weeks post immunization and assessed by ELISA for SARS-CoV-2 RBD-specific IgG1 (**a**) and IgG2a (**b**) titers. **c** Titer ratios of IgG2a to IgG1 were calculated. mRNA-RBD-L indicates the low dose (2 μg). mRNA-RBD-H indicates the high dose (15 μg). Data are means ± SEM (standard error of the mean). Comparisons were performed by Student's t-test (unpaired, two-tailed). Placebo animals = black circles; mRNA-RBD-L vaccinated animals = blue triangles; mRNA-RBD-H vaccinated animals = red squares; dotted line = the limit of detection. Data are one representative result of three independent experiments. Source data are provided as a Source Data file.

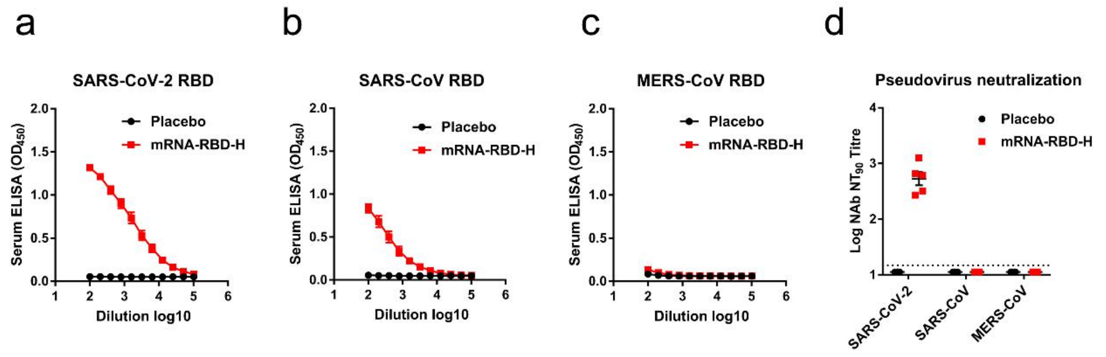

Supplementary Fig. 3 | Cross-reactivity of mRNA-RBD-induced sera to SARS-CoV and MERS-CoV. **Related to Fig. 2.** Groups of BALB/c mice (n=6) were immunized with a single injection of mRNA-RBD or placebo via the i.m. route. Sera at four weeks post immunization were collected. **a**-**c** ELISA analyses of binding of immunized sera on captured SARS-CoV-2 RBD (**a**), or cross-binding on captured SARS-CoV RBD (**b**) and MERS-CoV RBD (**c**). **d** Pseudovirus-neutralizing antibodies in sera against SARS-CoV-2, SARS-CoV and MERS-CoV were measured as NT_{90} titers. mRNA-RBD-H indicates the high-dose vaccine (15 µg). Data are means ± SEM (standard error of the mean). Placebo animals = black circles; mRNA-RBD-H vaccinated animals = red squares; dotted line = the limit of detection. Data are one representative result of three independent experiments. Source data are provided as a Source Data file.

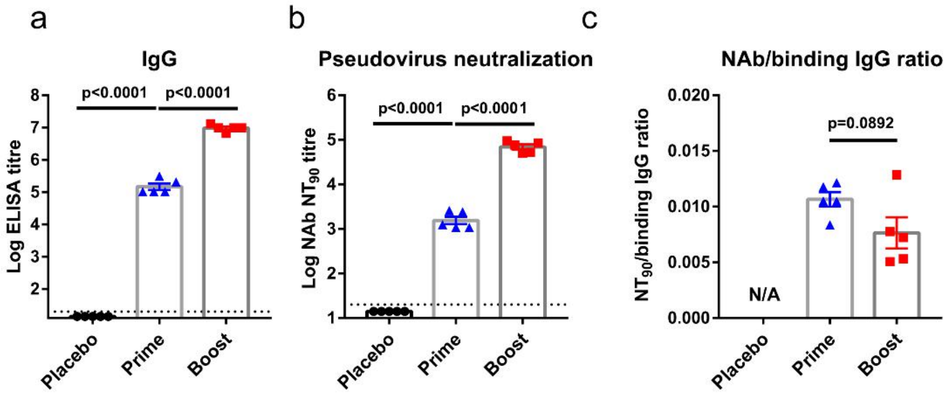

Supplementary Fig. 4 | Proportion analysis of neutralizing antibody to binding antibody elicited by mRNA-RBD-H prime and boost immunization regimens. BALB/c mice (n=5) were immunized i.m. with mRNA-RBD-H (15 μg) or a placebo. Boost group mice were boosted with an equivalent dose four weeks later. Serum was collected at eight weeks post immunization. RBD-specific IgG titers and pseudovirus-neutralizing antibodies were measured as shown in (**a**) and (**b**), respectively. **c** Titer ratio of neutralizing antibody to RBD-binding antibody was calculated. Data are means ± SEM (standard error of the mean). Comparisons were performed by Student's t-test (unpaired, two-tailed). Placebo animals = black circles; one injection-animals = blue triangles; two injections-vaccinated animals = red squares; dotted line = the limit of detection. Data are one representative result of two independent experiments. Source data are provided as a Source Data file.

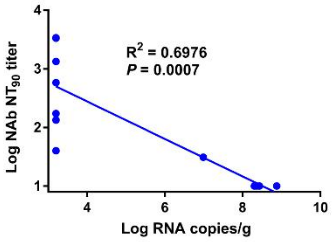

Supplementary Fig. 5 | Immune correlates of protection against SARS-CoV-2 in hACE2 transgenic mice. Related to Fig. 3. Correlation of viral loads in mouse lungs and protective efficacy by neutralizing antibody NT_{90} titers. The hACE2 mice receiving one or two doses of mRNA-RBD-H or placebo, and whose lung viral RNA loads following SARS-CoV-2 infection were determined (n=4 per group), were included in this analysis. The P and R^2 values reflect Spearman rank-correlation tests.

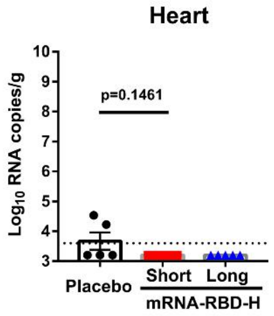

Supplementary Fig. 6 | Virus titers in heart. Related to Fig. 4. In the immunization and passive transfer study, heart tissues of challenged hACE2 transgenic mice (n=5) were collected at 5 dpi and were subjected to detection of viral RNAs load. Data are means ± SEM (standard error of the mean). Comparisons were performed by Student's t-test (unpaired, two-tailed). Placebo animals = black circles; animals for long term study = blue triangles; animals for short term study = red squares; dotted line = the limit of detection. Data are one representative result of three independent experiments. Source data are provided as a Source Data file.

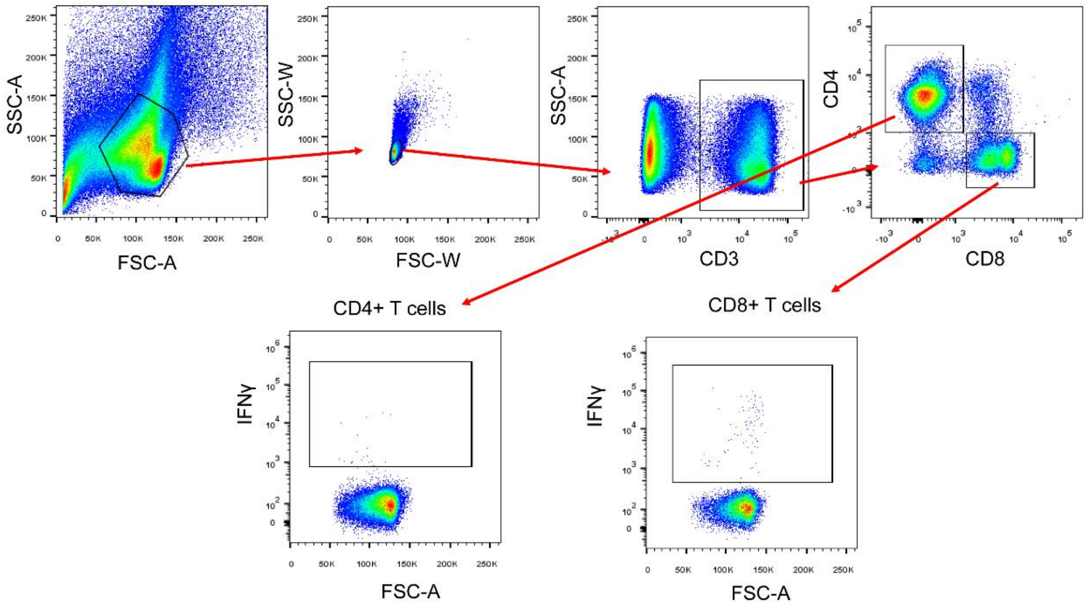

Supplementary Fig. 7 | Gating strategy of intracellular staining flow cytometry. Stimulated cells were stained and analyzed by flow cytometry. The Sample was progressively gated to identify single cells, lymphocytes, live CD3+ T cells, and CD4+ or CD8+ T cells as shown in the top row. Within CD4+ or CD8+ T cells, the gate for IFNγ was created (bottom).
