# Supplementary material for: A single-dose mRNA vaccine provides a long-term protection for hACE2 transgenic mice from SARS-CoV-2
Source: Nat Commun. 2021 Feb 3;12:776. doi: 10.1038/s41467-021-21037-2 (PMC7858593; doi:10.1038/s41467-021-21037-2)
Supplement: Supplementary file 3 — Reporting Summary [file 41467_2021_21037_MOESM3_ESM.pdf]

nature research

Corresponding author(s): Jinghua Yan

Last updated by author(s): Dec 22, 2020

Reporting Summary

Nature Research wishes to improve the reproducibility of the work that we publish. This form provides structure for consistency and transparency in reporting. For further information on Nature Research policies, see our Editorial Policies and the Editorial Policy Checklist.

Statistics

For all statistical analyses, confirm that the following items are present in the figure legend, table legend, main text, or Methods section.

n/a	Confirmed	
[ ]	[x]	The exact sample size (*n*) for each experimental group/condition, given as a discrete number and unit of measurement
[ ]	[x]	A statement on whether measurements were taken from distinct samples or whether the same sample was measured repeatedly
[ ]	[x]	The statistical test(s) used AND whether they are one- or two-sided<br>*Only common tests should be described solely by name; describe more complex techniques in the Methods section.*
[x]	[ ]	A description of all covariates tested
[x]	[ ]	A description of any assumptions or corrections, such as tests of normality and adjustment for multiple comparisons
[ ]	[x]	A full description of the statistical parameters including central tendency (e.g. means) or other basic estimates (e.g. regression coefficient) AND variation (e.g. standard deviation) or associated estimates of uncertainty (e.g. confidence intervals)
[ ]	[x]	For null hypothesis testing, the test statistic (e.g. *F*, *t*, *r*) with confidence intervals, effect sizes, degrees of freedom and *P* value noted<br>*Give P values as exact values whenever suitable.*
[x]	[ ]	For Bayesian analysis, information on the choice of priors and Markov chain Monte Carlo settings
[x]	[ ]	For hierarchical and complex designs, identification of the appropriate level for tests and full reporting of outcomes
[x]	[ ]	Estimates of effect sizes (e.g. Cohen's *d*, Pearson's *r*), indicating how they were calculated

Our web collection on statistics for biologists contains articles on many of the points above.

Software and code

Policy information about availability of computer code

Data collection	HALO v3.1.1076.379, BD FACSDiva Software 8.0.3, Immuno Capture 6.5.0
Data analysis	GraphPad Prism version 7, FlowJo 7.6.1

For manuscripts utilizing custom algorithms or software that are central to the research but not yet described in published literature, software must be made available to editors and reviewers. We strongly encourage code deposition in a community repository (e.g. GitHub). See the Nature Research guidelines for submitting code & software for further information.

Data

Policy information about availability of data

All manuscripts must include a data availability statement. This statement should provide the following information, where applicable:
- Accession codes, unique identifiers, or web links for publicly available datasets
- A list of figures that have associated raw data
- A description of any restrictions on data availability

The data supporting the findings of this study are available within this paper or are available from the corresponding author upon reasonable request. Source data are provided with this paper

Field-specific reporting

Life sciences study design

All studies must disclose on these points even when the disclosure is negative.

Sample size	For animal study, sample size were selected based on mean and standard deviations observed in our previous vaccine studies in these animal models, and ability to detect statistical differences between experimental groups of this size https://doi.org/10.1128/JVI .01722-17) Additionally, previous reports relevant to our study have used similar group sizes with comparable significance of the results (https://doi.org/10.1038/s41586-020-2622-0).<br>In vitro studies were performed with replicates and repeated several times based on our previous experience. (https://doi.org/10.1016/ j.cell.2020.06.035)
Data exclusions	No data was excluded.
Replication	All experiments in the study were successfully replicated at least 2-3 times. The number of replicates is described and /or shown for each dataset, and supports the reproducibility of the findings.
Randomization	For experiments involving mice, animals with the same gender, similar age and weight are randomly assigned to each group. For in-vitro study, randomization is irrelevant.
Blinding	One operator prepared vaccine samples. Different operators were blinded to group allocation for vaccination and collection of specimens from animals. The investigators were also blinded to group allocation during data acquisition and analysis such as the measurements of serum IgG, NAb titers, lung viral RNA titers and splenic cellular immune response.

Reporting for specific materials, systems and methods

We require information from authors about some types of materials, experimental systems and methods used in many studies. Here, indicate whether each material, system or method listed is relevant to your study. If you are not sure if a list item applies to your research, read the appropriate section before selecting a response.

Materials & experimental systems

n/a	Involved in the study	
☐	☒	Antibodies
☐	☒	Eukaryotic cell lines
☒	☐	Palaeontology and archaeology
☐	☒	Animals and other organisms
☒	☐	Human research participants
☒	☐	Clinical data
☒	☐	Dual use research of concern

Methods

n/a	Involved in the study	
☒	☐	ChIP-seq
☐	☒	Flow cytometry
☒	☐	MRI-based neuroimaging

Antibodies

Antibodies used	HRP-labeled Goat Anti-mouse IgG (Santa Cruz, Cat: sc-2005, Lot: D2915) 1 in 5000 µl,<br>HRP-labeled Goat Anti-mouse IgG (Abcam, Cat: ab97265, Lot: GR3195192-8) 1 in 10000 µl,<br>HRP-labeled Goat Anti-mouse IgG1 (Abcam, Cat: ab97240, Lot: GR168230-1) 1 in 10000 µl, HRP-labeled Goat Anti-mouse IgG2a (Abcam, Cat: 97245, Lot: GR174542-3) 1 in 10000 µl,<br>PE anti-mouse CD3 antibody (Biolegend, Clone: 17A2, Cat: 100206, Lot: B277602) 0.25 in 50 µl,<br>Percp-cy5.5 anti-mouse CD8a antibody (Clone 53-6.7, Biolegend, Cat: 100734, Lot: B289962) 1 in 50 µl,<br>APC-cy7 anti-mouse CD4 antibody (Clone RM4-5, Biolegend, Cat: 100526, Lot: B282355) 1 in 50 µl,<br>FITC anti-mouse IFN-γ antibody (Clone XMG1.2, Biolegend, Cat: 505806, Lot: B243512) 1 in 50 µl,<br>Rabbit MAb anti-SARS-CoV-2 Nucleocapsid antibody (Clone 019, SinoBiological, Cat: 40143-R019, Lot: HA14FE1703) 1 in 100 µl,<br>HPR-labeled Goat Anti-rabbit IgG (H+L) (Abways, Cat: ab0101, Lot: F300405) 1 in 500ul.
Validation	All commercially available antibodies used are validated.<br><br>ELISA<br>HRP-labeled Goat Anti-mouse IgG (Abcam, Cat: ab97265): specific for mouse IgG, suitable for ICC, IHC-P, ELISA and WB. Manufacturer website provides the datasheet “Goat Anti-Mouse IgG Fc (HRP) ab97265”. The datasheet states this antibody was used in 23 citations.<br>HRP-labeled Goat Anti-mouse IgG1 (Abcam, Cat: ab97240): specific for mouse IgG1, suitable for ICC, ELISA, IHC-P and WB. Manufacturer website provides the datasheet “Goat Anti-Mouse IgG Fc (HRP) ab97240”, The datasheet states this antibody was used in 49 citations.<br>HRP-labeled Goat Anti-mouse IgG2a (Abcam, Cat: ab97245): specific for mouse IgG2a, suitable for IHC-P, ELISA, WB and ICC. Manufacturer website provides the datasheet “Goat Anti-Mouse IgG Fc (HRP) ab97245”, The datasheet states this antibody was used in 24 citations.

Western Blot
HRP-labeled Goat Anti-mouse IgG (Santa Cruz, Cat: sc-2005): specific for mouse IgG, suitable for WB. Manufacturer website provides the datasheet “goat anti-mouse IgG-HRP: sc-2005”, The datasheet states this antibody was used in 927 citations.

Flow Cytometry
PE anti-mouse CD3 antibody (Clone 17A2, Biolegend, Cat: 100206): Reactivity, Mouse; Application, Flow cytometry (Quality tested); this antibody was used in 48 citations.
Percp-cy5.5 anti-mouse CD8a antibody (Clone 53-6.7, Biolegend, Cat: 100734): Reactivity, Mouse; Application, Flow cytometry (Quality tested); this antibody was used in 81 citations
APC-cy7 anti-mouse CD4 antibody (Clone RM4-5, Biolegend, Cat: 100526): Reactivity, Mouse; Application, Flow cytometry (Quality tested); this antibody was used in 25 citations
FITC anti-mouse IFN-γ antibody (Clone XMG1.2, Biolegend, Cat: 505806): Reactivity, Mouse; Application, Intracellular staining for Flow cytometry (Quality tested); this antibody was used in 53 citations

IHC
Rabbit MAb anti-SARS-CoV-2 Nucleocapsid antibody (Clone 019, Sino Biological, Cat: 40143-R019): specific for SARS-CoV-2 nucleocapsid protein, suitable for WB, ELISA. Manufacturer website states that IHC application has not been validated. However, this antibody used for IHC assays was reported (https://doi.org/10.1016/j.ekir.2020.04.002). Manufacturer website provides the datasheet “SARS-CoV-2 (2019-nCoV) Nucleocapsid Antibody, Rabbit MAb”, The datasheet states this antibody was used in 8 citations.
HRP-labeled Goat Anti-rabbit IgG (H+L) (Abways, Cat: ab0101): Reactivity: Rabbit; Application: WB and IHC. Manufacturer website provides the datasheet “Goat Anti-Rabbit IgG (H+L) HRP”.

Eukaryotic cell lines

Policy information about cell lines

Cell line source(s)	HEK293T cells: ATCC, CRL-3216;<br>Vero E6 cells: ATCC, CRL-1586.<br>Huh7 cells was purchased from Institute of Basic Medical Sciences, Chinese Academy of Medical Sciences (Cat# 3111C0001CCC000679; RRID: CVCL_0336).
Authentication	The cell lines were not authenticated since they were purchased commercially and are not commonly misidentified.
Mycoplasma contamination	The cells were not tested for mycoplasma contamination.
Commonly misidentified lines<br>(See ICLAC register)	The cell lines used in this study do not appear on the ICLAC register.

Animals and other organisms

Policy information about studies involving animals; ARRIVE guidelines recommended for reporting animal research

Laboratory animals	Specific pathogen-free (SPF) female BALB/c and C57BL/6 mice aged 6-8 weeks were purchased from Beijing Vital River Animal Technology Co., Ltd. (Beijing, China). The female hACE2 transgenic mice aged 6-8 weeks were kindly provided by Professor Zhengli Shi from Wuhan Institute of Virology, CAS (Wuhan, China).
Wild animals	Study did not involve mild animals.
Field-collected samples	Study did not involve samples collected from the field.
Ethics oversight	All of the animal experiments were reviewed and approved by the Committee on the Ethics of Animal Experiments of the Institute of Microbiology, Chinese Academy of Sciences.

Note that full information on the approval of the study protocol must also be provided in the manuscript.

Flow Cytometry

Plots

Confirm that:

- [x] The axis labels state the marker and fluorochrome used (e.g. CD4-FITC).
- [x] The axis scales are clearly visible. Include numbers along axes only for bottom left plot of group (a 'group' is an analysis of identical markers).
- [x] All plots are contour plots with outliers or pseudocolor plots.
- [x] A numerical value for number of cells or percentage (with statistics) is provided.

Methodology

Sample preparation	Mouse spleens were removed from vaccinated C57BL/6 mice at four weeks post immunization, and splenocytes were

	isolated and were added to the plate (1 × 10^6 cells/well). The cells were stimulated with the peptide pool (2 μg/ml of individual peptide) for 4 h. Then the cells were incubated with Golgiplug (BD Biosciences) for an additional 12 h at 37°C. The cells were harvested and stained with anti-CD3, anti-CD4, and anti-CD8α surface markers (Biolegend). The cells were subsequently fixed and permeabilized in permeabilizing buffer (BD Biosciences) and stained with anti-IFN-γ (Biolegend).
Instrument	BD FACSAria III flow cytometer
Software	FlowJo 7.6.1
Cell population abundance	At least one million splenocytes per sample were used for intracellular cytokine staining, and at least 200,000 events were collected for each sample on flow cytometer. The positive population was identified using similarly stained but vaccine un-transfected cells to set the positive cut-off.
Gating strategy	The gating strategy used was as to first gate on singlets. Then CD3+ cells were gated and from that population CD4+ and CD8 + T cells were gated. The IFN-γ cytokine was gated from each of the CD4 and CD8 T cell populations.

☒ Tick this box to confirm that a figure exemplifying the gating strategy is provided in the Supplementary Information.
